# Hypertrophic cardiomyopathy risk stratification based on clinical or dynamic electrophysiological features: two sides of the same coin

**DOI:** 10.1093/europace/euad072

**Published:** 2023-03-21

**Authors:** Arunashis Sau, Fu Siong Ng

**Affiliations:** National Heart and Lung Institute, Imperial College London, Hammersmith Campus, Du Cane Road, London W12 0NN, UK; Department of Cardiology, Hammersmith Hospital, Imperial College Healthcare NHS Trust, 72 Du Cane Road, W12 0HS, London, UK; National Heart and Lung Institute, Imperial College London, Hammersmith Campus, Du Cane Road, London W12 0NN, UK; Department of Cardiology, Hammersmith Hospital, Imperial College Healthcare NHS Trust, 72 Du Cane Road, W12 0HS, London, UK; Department of Cardiology, Chelsea and Westminster Hospital NHS Foundation Trust, 369 Fulham Road, SW10 9NH, London, UK

## Abstract

**Graphical Abstract:**
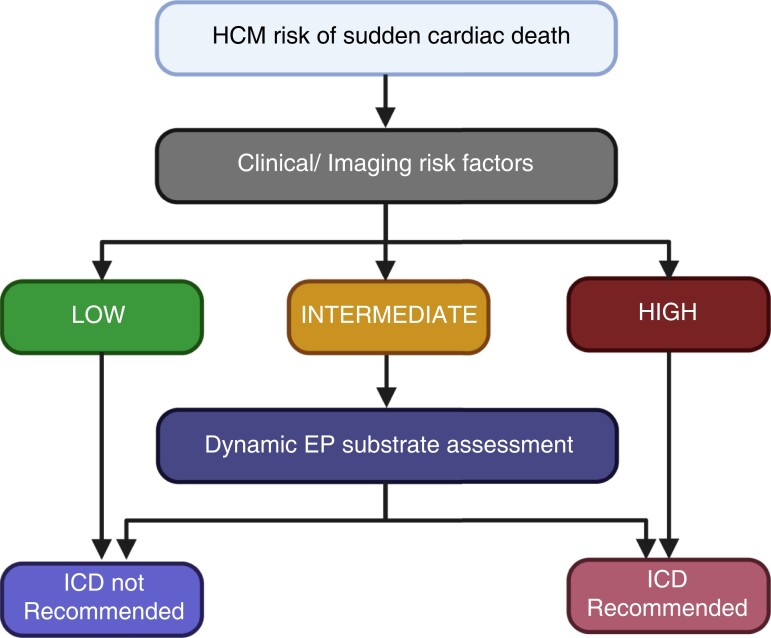

**This editorial refers to ‘Should lethal arrhythmias in Hypertrophic Cardiomyopathy be predicted using non-electrophysiological methods?’, by R. Saumarez *et al.*, https://doi.org/10.1093/europace/euad045**.

Hypertrophic cardiomyopathy (HCM) is the commonest inherited cardiac condition, with a prevalence of between 1:200 and 1:500 in adults.^1^ Recognition of HCM is increasing with improved access to cardiovascular imaging and family screening. Hypertrophic cardiomyopathy can increase the risk of ventricular arrhythmias and sudden cardiac death (SCD), though event rates overall are low.^2^ Risk stratification for SCD remains a significant challenge, and accurate methods to predict individuals with HCM at highest risk of SCD remain elusive. The current paradigm, in general, involves the use of clinical risk factors and imaging parameters to assess risk of SCD.^3,4^ Depending on the specific clinical guidelines used, these may include age, left ventricular (LV) outflow gradient, maximal LV wall thickness, left atrial diameter, family history of sudden cardiac death, degree of left ventricular hypertrophy, unexplained syncope, apical aneurysm, reduced LV ejection fraction, non-sustained ventricular tachycardia, and extensive late gadolinium enhancement on cardiac magnetic resonance imaging.

In general, if a high-risk threshold is reached, a primary prevention implantable cardioverter defibrillator (ICD) would be recommended. However, despite these measures, the majority of SCD occurs in the low-risk cohorts, demonstrating the imperfect nature of the current risk stratification approach.^5^ Additionally, given the not insignificant long-term incidence of lead-related complications,^6^ ICD implantation should not be taken lightly. Accurate selection of patients who are most likely to benefit from an ICD is therefore paramount.

Saumarez *et al*.^7^ seek to challenge the current paradigm and explore the potential benefits of an electrophysiology (EP)-based approach to HCM risk stratification. There is a clear and sound rationale for focusing on the electrophysiological substrate, rather than clinical features, as SCD in HCM is often due to ventricular arrhythmias that are caused by abnormal ventricular electrophysiology. Their EP-first approach is based on the concept of paced ventricular electrogram fractionation (PEFA), first described over 30 years ago.^8^ Briefly, this involves four EP catheters placed in different right ventricular (RV) locations, used to pace, and record intracardiac electrograms. Paced extra-stimuli are used to provoke delayed potentials (fractionated electrograms) with progressively shortened pacing coupling intervals. Paced ventricular electrogram fractionation aims to quantify SCD risk through analysis of the change in electrogram morphology in response to the pacing protocol. Five previously published studies of HCM SCD risk stratification were analysed, four based on clinical/imaging risk factors and one using an EP-based approach (PEFA). They estimated population statistics using bootstrapping or Monte Carlo simulation and found the EP-based approach had a mean receiver operating characteristic area under the curve (ROCAUC) of 0.89 compared to 0.67–0.72 for the risk factor methods. Importantly, the EP-based approach was found to detect 90% of events with a relatively low false positive rate of 20%.

The occurrence of ventricular arrhythmia in HCM may be due to several potential mechanisms including myocyte disarray causing re-entry cycles^9^ and abnormal myocyte calcium handling. Therefore, risk stratification based on clinical factors and imaging do not address the underlying causative factors for SCD. On the other hand, an EP-based risk stratification tool has the advantage of being closely linked mechanistically to re-entrant mechanisms that may lead to ventricular arrhythmias and SCD. Indeed, Saumarez *et al*. show the markedly superior predictive ability of the EP approach.

Another advantage of an EP-based approach to risk stratification is the ability to assess the response to a dynamic extrinsic stressor. Dynamic stressors can include exercise and ventricular pacing and may reveal an underlying propensity to ventricular arrhythmia that may not be clearly evident at rest. Ventricular arrhythmias occurring on exercise in particular are associated with an increased risk of SCD in HCM.^10^ The EP-based approach discussed by Saumarez *et al*. uses pacing at varied coupling intervals, which has a similar effect of perturbing the underlying substrate to unmask any arrhythmic tendency. Highlighting the importance of dynamic assessment of the underlying substrate, electrocardiographic imaging (ECGI) following exercise was also previously found to highlight those at risk of ventricular arrhythmia.^11^

Given the powerful predictive value of EP-based risk stratification demonstrated by Saumarez *et al*. and the strong links to the underlying mechanistic basis for ventricular arrhythmia, an EP-based approach would appear ideal for HCM risk evaluation. However, the invasive nature of this procedure provides a significant challenge. Given the growing HCM population,^12^ many of whom will be asymptomatic individuals diagnosed through family screening, invasive testing for all does not seem appropriate, and would neither be practical nor feasible. The need for serial repetition of risk assessment as HCM the phenotype evolves is an additional important consideration that argues against invasive risk stratification.^3^ Lastly, given the multitude of factors that may affect results of electrophysiology studies (EPS) for other conditions, including autonomics, the reproducibility of EP-based risk assessment for HCM is unclear.

The imperfect nature of both the risk factor-based approach and EP-based approach begs the question if any other approach is available. Artificial intelligence (AI) has had an exponential growth in the last 10 years and has been used for both diagnostic and predictive applications. Applied to the ECG, AI can accurately diagnose heart failure and HCM, predict incident atrial fibrillation, and predict mortality.^13–16^ Early work suggests the potential for AI to be applied to specific cohorts (such as HCM) for risk prediction.^17–19^ However, the results of these studies are modest at best and lacking key features, including validation in appropriate external populations. Artificial intelligence holds promise for the future but is not currently ready for use in HCM risk stratification.

Until then, the optimal approach for risk stratification for HCM is likely to involve a combination of both clinical and dynamic electrophysiological features. A pragmatic approach would be to use the current clinical risk factor/imaging-based approach to identify individuals at the two ends of the spectrum, at the highest and lowest risk, to recommend an ICD or reassure, respectively. For individuals at intermediate risk (e.g. 4–6% risk of SCD over 5 years^4^), it may then be helpful to perform dynamic interrogation of the EP substrate, using methods such as PEFA, to guide ICD implantation decisions in these borderline cases. The authors should be congratulated for showing the clear utility of using dynamic EP substrate assessment for risk stratification in HCM.
